# Comparison of classic peribulbar anesthesia and new entry point (single percutaneous injection technique) in vitroretinal surgery

**DOI:** 10.4103/1658-354X.65130

**Published:** 2010

**Authors:** Tarek M. El Said, Mamdouh Mahmoud Kabeel

**Affiliations:** *Department of Anesthesiology, Al-Azhar University, Egypt*; 1 *Department of Ophthalmology, Tanta University, Egypt*

**Keywords:** *Peribulbar anesthesia*, *single percutaneous injection technique*, *vitroretinal surgery*

## Abstract

**Purpose:**

To describe the method that seeks to improve the administration of regional anesthesia for vitroretinal surgery avoiding the risk of potential complications associated with other techniques through comparison of safety and efficacy of classic peribulbar anesthesia versus single percutaneous technique using a prospective, randomized clinical trial.

**Materials and Methods:**

One hundred patients were randomized to classic peribulbar and single percutaneous peribulbar technique after informed consent. Pain during administration of anesthesia, during surgery was graded on a visual analogue pain scale and compared for both techniques. Globe akinesia, analgesia and IOP measurements before and after administration of anesthesia, detection of distribution of local anesthetic agent by ultrasound scanning and complications related were also compared.

**Results:**

Twenty out of 50 (40%) patients of group 1(classic pirebulbar) and 36/50 (72%) of group II (single percutaneous technique) experience no pain during administration of anesthesia. Scores for globe akinesia and anesthesia were less satisfactory in group 1 and supplemental blocks required in 8% of the patients while in group II all of the patients (100%) showed proper globe akinesia and anesthesia. There were significant elevation in mean IOP following injection in both groups and the incidence of subconjunctival haemorrhage, chemosis and echymosis were more frequent in group 1.

**Conclusion:**

Single percutaneous peribulbar technique proved to be a safe and efficient technique that offers excellent anesthesia and akinesia with less complication for various ophthalmic procedures

## INTRODUCTION

Peribulbar anesthesia for intraocular surgery has gained in popularity over the last few years. It is relatively safe, easy to perform, provides effective ocular akinesia and anesthesia. Peribulbar anesthesia is therefore a near-ideal anesthetic technique for ophthalmic surgery. Unfortunately, it tends to produce some risks,[1] for example, conjunctival chemosis, subconjunctival hemorrhage, risk of injury of intraorbital structures. So, various modifications have been devised over the last two decades to reduce the complications associated with the technique. Low-volume two-injection technique gives acceptable conditions for ophthalmic operation. However, it is suggested that percutaneous single injection is safer in eye surgery.[2,3] We conducted randomized single-blind study to evaluate and compare single injection percutaneous peribulbar technique (as suggested by Leonardo Rizzo)[4] with classic peribulbar technique for globe/lid akinesia, globe anesthesia and intraocular pressure (IOP). Also we compared the distribution of local anesthetic by ultrasound scanning,[5] chemosis, block-related complication, patient‘s discomfort and overall surgeon‘s satisfaction with operating conditions and effectiveness of the block between the two techniques.

## MATERIALS AND METHODS

One hundred patients were randomized into two groups scheduled for vitroretinal surgery, classic peribulbar anesthesia group (group 1, *n* = 50) and single percutaneous peribulbar anesthesia (group 2, *n* = 50). After approval from local managing committee, informed consent was obtained from the patients. Preoperative screening was done and patients who were having active infection or evidence of infection were not included, other exclusion criteria were sensitivity to local anesthetic drugs, history of convulsion or epilepsy, mental instability, previous intraocular injury or surgery and patients with posterior syneachia. All patients were premedicated with midazolam IV (0.02 mg/kg) 5 minutes before the block.

All the patients were monitored and had intravenous access. Patients were connected to monitors for blood pressure, electrocardiogram respiratory rate, pulse oximetry and nasal catheter for oxygen at a rate of 3-5 Litre/min.

In the first group (classic peribulbar anesthesia), the injection containing local anesthetic 6–8 mL mixture containing 2% xylocaine and 0.5 marcaine in combination with 150 IU hyaluronidase was given at the junction of the lateral third and medial two-thirds of the lower orbital margin. The injection was given with a 25-gauge, 25-mm cutting bevel disposable needle; external compression from the Honan balloon was applied for 5 minutes after injection.

In the second group (single percutaneous peribulbar anesthesia), the injections were given with a 25-gauge, 16-mm bevel disposable needle. The anesthetic was a 5–6 mL mixture containing 2% xylocaine and 0.5 marcaine in combination with 150 IU hyaluronidase on the basis of dimension of the eye socket. The patients were asked to move their eye so as to expose the area to be injected at 10 a.m. for the right eye and to 2 p.m. for the left eye . The injection site was percutaneous and limited superiorly from inferior lacrimal canaliculus, median from lateral margin of nose, laterally from imaginary perpendicular line that joins inferior lacrimal papilla to inferior margin of orbit and inferiorly from inferior margin of orbit.

The needle was advanced percutaneously in an antero-posterior direction for half of its length (never more than 10 mm) and later obliquely in the direction of the optical foramen until the needle was on the same plane of the bony margin of orbit. After aspiration, the anesthetic was injected in approximately 30 s. An initial transient fullness in the supero-internal region of superior lid was predictive of successful blockade. After the needle was removed, external compression from the Honan balloon was applied for 5 minutes after injection.

Assessment of eye movements was done after 10 minutes for all patients because it is the maximum fixation time for the mixture of local anesthesia used. The patients were asked to grade the pain they felt on a linear scale of 0–4 (no pain = grade 0, mild pain = grade 1, moderate pain = grade 2, severe pain = grade 3, maximum pain imaginable = grade 4). Patients were asked to grade separately the pain during administration of anesthesia and pain during surgery. All patients had their IOP measured immediately before the peribulbar anesthesia with tonopen XL (Medtronic, Minneapolis, USA) in both the groups and 5 minutes after injection.

Akinesia was assessed by subjective scale graded as complete movement remaining, partial movement remaining, and no movement, and a point scale of 0, 1, and 2, respectively.

Global anesthesia (feeling pain on touch) was assessed on a 0–2 scale where 0 = no anesthesia, 1 = partial but acceptable anesthesia and 2 = complete anesthesia. Supplemental blocks were given when akinesia and anesthesia were inadequate. The sites of supplementary injections in the first group are supero-medial for superior or medial movement and infero-temporal for inferior lateral movement, whereas in the second group, the site of supplementary injection is the same as before. All supplements were given after 10 minutes by a needle which was of the same type as that used for the primary injection.

Detection of distribution of local anesthetic inside the blocked eye was done after 5 and 10 minutes through using high-resolution B-scanning (OTI, ub2, 08822, Ontario, Canada). It is an acoustic wave that consists of an oscillation of particles. By definition, U/S waves have a frequency greater than 20 kHz which render them inaudible; these very high frequencies used in ophthalmology produce short wavelengths in the range of 0.2 mm. These very short wavelengths allow sufficient resolution of the minute structures in the eye and orbit and this helped us to detect precisely the location and the distribution pass of the local anesthetic fluids in the retro-ocular tissue.

By using the B-scan for both the vertical and horizontal dimensions of the screen (to indicate the exact location and configuration of any abnormal collection rather than the normal echogenic pattern of the retro-orbital fat), the anesthetic fluid was identified as a not well-circumscribed low-reflective, dark void area surrounded by the highly reflective orbital fat or the extra ocular muscle.

Degree of chemosis and subconjunctival hemorrhage were noted after 10 minutes on a subjective scale (none = 0, mild = 1, moderate = 2 and severe = 3). Immediate complication due to the block, for example, hematoma, echymosis, retrobulbar hemorrhage, were also recorded. Long-term complications were not reported in this study.

### Statistical analysis

The collected data were grouped, tabulated and statistically analyzed using SPSS software program. For quantitative variable, the mean and standard deviation were calculated and the difference between groups was tested using unpaired t test . For categorical variables, the number and percent were calculated and the difference statistically analyzed using chi-square or Fisher exact test as appropriate. The level of significance was adopted at *P* < 0.05.

## RESULTS

The various grades of pains during anesthesia are depicted in Table 1. Chi-square test showed that there was a significant difference between both the groups with regard to pain on anesthesia (*P* < 0.001). Table 2 shows the various grades of pain surgery in both the groups.

**Table 1:** Pain during anesthesia

Grade	Classic peribulbar technique (group 1 *n* = 50)	Single percutaneous peribulbar technique (group 2 *n* = 50)	χ^2^	*P*
0 (No pain)	20	36	10.39	0.001*
1 (Mild pain)	25	12		
2 (Moderate)	4	2		
3 (Severe)	1	0		
4 (Maximum imaginable)	0	0		

For statistical analysis grades 1, 2, 3, and 4 were grouped;

* *P* <0.05 significant.

**Table 2:** Pain during surgery

Grade	Classic peribulbar technique (group 1 *n* = 50)	Single percutaneous peribulbar technique (group 2 *n* = 50)	*P*
0 (No pain)	49	50	1.000
1 (mild pain)	1	0	
2 (Moderate)	0	0	
3 (Severe)	0	0	
4 (Maximum imaginable)	0	0	

Assessment of akinesia and analgesia are summarized in Table 3. Exactly 100% of the patients had adequate globe anesthesia, but in group I (classic peribulbar technique), these results were obtained after supplemental block which was required in 8% of the patients. Globe akinesia was better in group 2 (single percutaneous technique) 100% versus 92% in group 1. Both the groups were equal with regard to globe akinesia after supplemental block in group 1.

**Table 3:** Akinesia and anesthesia

Akinesia and anesthesia	Grade	Classic peribulbar technique (group 1 *n* = 50)	Single percutaneous peribulbar technique (group 2 *n* = 50)	*P*
Globe akinesia	0	0	0	0.059
	1	4	0	
	2	46	50	
Globe anesthesia	0	0	0	1.000
	1	0	0	
	2	50	50	
Supplemental block		4	0	0.059

For statistical analysis grades 0 and 1 were grouped

Following injection, there was a significant rise (*P* < 0.001) in IOP (mean postinjection pressure 38.9 ± 1.1) in group I with six patients having IOPs greater than 50 mmHg. After 5 minutes of compressions, the IOPs did not differ significantly from the preinjection level. In group II, there was a significant rise in IOP (*P* < 0.001) with a mean postinjection pressure of 32.4 ± 1.2 mmHg for which one patient had the IOP greater than 50 mmHg. IOPs after 5 minutes of application of the Honan balloon not differ significantly from the preinjection level [Table 4].

**Table 4:** Pre- and post-intraocular pressure with standard deviations

Intraocular pressure	Classic peribulbar technique (group 1 *n* = 50)	Single percutaneous peribulbar technique (group 2 *n* = 50)	*t*	*P*
Preinjection	17.2 ± 3.1	17.8 ± 2.3	1.099	0.274
Postinjection	38.9 ± 1.1	32.4 ± 1.2	28.234	0.001*
Post-Honan	18 ± 2.5	17 ± 1.4	2.468	0.015

* *P* <0.05 significant.

**Figure 1:**
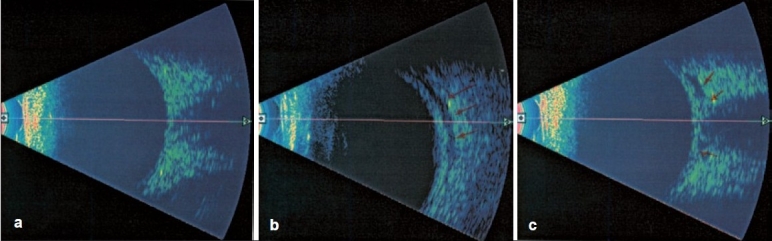
(a) The ultrasound of the eye preinjection with axial scan pass through the optic nerve. (b) The ultrasound 5 minutes after injection of the local anesthetics in the longitudinal axial plan at 6 a.m. of the globe, the red arrows indicate the hypo-echogenic track of the tracked local anesthetics in the subtenon space. (c) The same globe 10 minutes after injection with axial plan through the optic nerve and the red arrows demonstrate the hypo-echogenic pattern around the optic nerve giving the characteristic T sign of perfusion of local anesthetics

**Figure 2:**
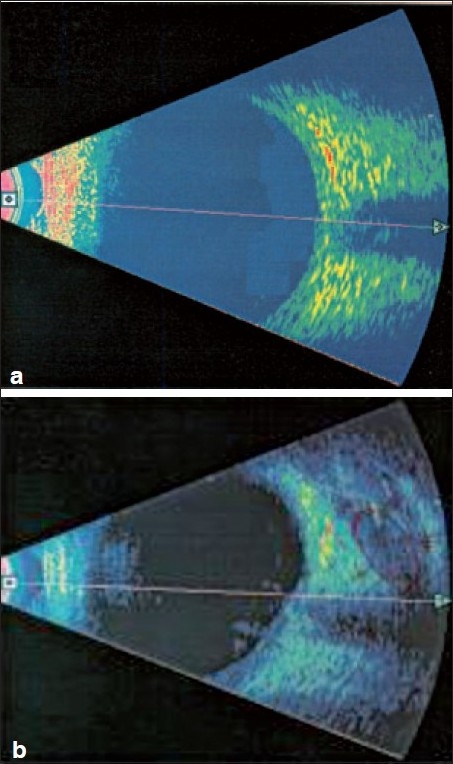
(a) The ultrasound axial scan through the optic nerve preinjection with homogenous echogenecity of retro orbital path around the optic nerve. (b) The ultrasound lower vertical scan 10 minutes after injection and the axial scan pass below the red ring that demonstrate the hypo-echogenic pattern of local anesthetic fluid collection in the inferior surface of the nerve which starts to track in the direction of the muscle cone

Localization of the peribulbar local anesthetic was initially indistinguishable and difficult but was subsequently observed in the orbital fat posterior to the globe. Ten minutes latter, the anesthetic fluid was identified in the cone mostly inferior to the nerve in group 1 (classic peribulbar) [Figure 1a and b]. In group 2 (single percutaneous technique), the fluid was tracked behind the globe in sub tenon capsule space to one side first of the optic nerve, then diffused posteriorly around the optic nerve and developed a characteristic T sign when the anesthetic fluid equally distributed around the optic nerve, and later distributed into the whole muscle cone. [Figure 2a–c]

Complications after administration of regional anesthetic in both the groups are described in Table 5; there are clinical differences between both the groups but are not statistically significant. More conjunctival chemosis (10%), various grades of subconjunctival hemorrhage (8%), echymosis (6%) and lower lid hemorrhage (4%) occurred in group 1 (classic peribulbar technique).

**Table 5:** Complication after administration of anesthesia

Complications	Grade	Classic peribulbar technique (group I *n* = 50)	Single percutaneous peribulbar technique (group II *n* = 50)	*P*
Subconjunctival hemorrhage	0	46	50	0.059
	1	3	0	
	2	1	0	
	3	0	0	
Chemosis	0	45	49	0.204
	1	5	1	
	2	0	0	
	3	0	0	
Echymosis		3	0	0.242
Lower lid hemorrhage		2	0	0.495
Retrobulbar hemorrhage		0	0	1.000
Globe perforation		0	0	1.000

There were no cases of perforation of the globe, retinal or optic nerve damage or orbital hematoma in both the groups. No operation had to be cancelled because of a problem with the rergional anesthetic block and none of the patients in the study needed general anesthesia.

## DISCUSSION

The delivery of single percutaneous peribulbar technique is effective and reliable in providing both analgesia and akinesia.[6] It appears to get closer to the characteristics of an ideal block than the classic peribulbar technique. It uses smaller injectate volume and had speed of onset comparable to the classic technique.

It avoids many of the complications associated with the classic technique. Another advantage of the single percutaneous technique is that it is usually associated with only minor discomfort, which may explain the excellent degree of patient acceptability.[7] Single percutaneous peribulbar technique was more comfortable for the patient at the time of anesthetic administration and during surgery. The patients‘ satisfaction is enhanced by warning them just before the injection that they might experience a mild sensation like a pressure or even mild discomfort. Seventy two percent of the patients in group 2 reported no pain during anesthetic administration and 100% reported no pain during surgery in comparison to 40 and 98%, respectively, of the patients in the classic peribulbar technique (group 1), which is statistically significant at the time of local anesthetic administration. One of the advantages of the single percutaneous technique is that the pain decreased with percutaneous short needle insertion.

In this study, the results showed the globe akinesia and globe anesthesia was better in Group 2. However, they were not statistically significant. In our experience, akinesia was always satisfactory in peribulbar anesthesia. Surgeons find it difficult to work without complete akinesia and anesthesia during vitroretinal surgery as reported by many authors. So, supplemental block in peribulbar anesthesia remains the major constraint of the technique. The reported incidence is between 5 and 63% in various studies.[8,9]

In this study, we have found significant difference in supplemental injections among the two techniques that we used. The supplemental injection was 10% in the first group which resulted in satisfactory painless operating conditions, whereas in the second group there was no supplemental block.

We agree with Ball *et al*.[3] that an adequate block can be achieved with a single peribulbar injection placed either inferotemporally (classic technique)[10] or medially (single percutaneous technique)[11] and there is no evidence that a second primary injection decreases the rate of supplemental injection required. We also therefore propose that a second primary peribulbar injection is unnecessary and many carry an increased risk of globe perforation. Therefore, we used second injection only when required.

We investigated the changes in IOP that follow peribulbar anesthesia, which were measured immediately after injection in both groups and 5 minutes after Honan balloon compression. There was a significant increase in IOP after injection with a mean postinjection pressure of 38.9 ± 1.1 and 32.4 ± 1.2 mmHg in groups 1 and 2, respectively, whereas after 5 minutes of compression, IOPs did not differ significantly from the preinjection level. So, the single percutaneous technique is more suitable for intraocular surgery in patients with compromised intraocular circulation.[12,13] Unfortunately, our results are not directly comparable with measurements of IOP that have been made as in a study of retrobulbar injection.[14] The explanation might be that the volumes (3–4 mL) of local anesthetic used in this study were smaller than those routinely used in peribulbar injections. However, our results were comparable with those of RoPo *et al*.,[15] who investigated the changes in IOP that follow peribulbar anesthesia (classic technique).

The sensitivity of the eyeball is provided by the ciliary nerves, which cross the episcleral space after they emerge from the globe. The fascial sheath of the eyeball (tenon‘s capsule) extends to rectus muscle sheath. This explains why the anesthetic is preferentially guided to this muscle sheath to produce good akinesia; also, the fascial sheath of the eyeball guides the injected solution to the lids, especially to the orbicularis muscle preventing blinking during surgery without performing any facial nerve block. This explains why single percutaneous technique is more effective than the classic peribulbar technique.

Anesthesia-related chemosis, subconjunctival hemorrhage, echymosis occurred mainly in the first group. No patient had globe injury, perforation or retrobulbar hemorrhage in both the groups. In the peribulbar anesthesia by a medial percutaneous single injection (group 2), infrequent complications were observed; this site of injection is relatively avascular, which may decrease the risk of hematoma.[16]

Indeed, we noted no orbital hematoma in this group. Because the insertion of the needle is limited to the anterior orbit, ophthalmic artery, optic nerve or retinal injury is unlikely.

Based on our results, we can conclude that the application of the single percutaneous technique provides a level of comfort during intraocular surgery. This technique is a simple and satisfactory alternative approach for ocular regional anesthesia. The advantages include decreased pain with percutaneous and short needle insertion, decreased volume of anesthetic, single rather than multiple punctures with less complication.
